# Serum Levels of Brain-Derived Neurotrophic Factor and Other Neurotrophins in Elite Athletes: Potential Markers of the Use of Transcranial Direct Current Stimulation in Sport

**DOI:** 10.3389/fspor.2021.619573

**Published:** 2021-04-12

**Authors:** Francesco Donati, Veronica Sian, Giorgia Morgan Biasini, Xavier de la Torre, Fabrizia Folchitto, Francesco Botrè

**Affiliations:** ^1^Laboratorio Antidoping, Federazione Medico Sportiva Italiana, Rome, Italy; ^2^REDs – Research and Expertise in anti-Doping Sciences, ISSUL – Institute of Sport Sciences, University of Lausanne, Lausanne, Switzerland

**Keywords:** brain-derived neurotrophic factors, neurotrophins, transcranial direct current stimulation, genetic polymorphisms, sport performance

## Abstract

Transcranial Direct Current Stimulation (tDCS) is a non-invasive brain stimulation that may enhance mental and physical performance in sports, representing a potential new form of doping (“brain doping” or “electromagnetic doping”). This study aims to identify diagnostic biomarkers for detecting the possible abuse of tDCS in sport. Brain-Derived Neurotrophic Factor (BDNF) and other neurotrophins (NT, such as beta nerve growth factor, NGF) were pre-selected as potential candidates since their serum values have been observed to change following tDCS. Neurotrophins were measured using ELISA assays in 92 serum samples collected from elite athletes, classified by sex (males = 74; females = 18), age (range 17–25 *n* = 27, 26–35 *n* = 36, and over 35 *n* = 14; age not known *n* = 15), type of sports practiced (endurance *n* = 74; power *n* = 18), and type of sample collection (“in competition” *n* = 24; “out of competition” *n* = 68). Single nucleotide polymorphisms (rs6265, rs11030099, and rs11030100) were genotyped on 88 samples to determine their influence on the analytes' basal levels. Athletes older than 35 presented higher BDNF values than younger individuals (*p* < 0.05). Samples collected “in competition” showed higher BDNF concentrations than those collected “out of competition” (*p* < 0.05). The studied polymorphisms appeared to affect only on proBDNF, not altering BDNF serum concentrations. NT-3 and NT-4 were poorly detectable in serum. Our results suggest that BDNF can be considered as a first biomarker to detect the abuse of tDCS in sport doping. Further studies are necessary to assess whether proBDNF and beta NGF can also be considered suitable biomarkers to detect the recourse to electromagnetic brain stimulation in sports, especially in the case their serum levels can be monitored longitudinally. To the best of our knowledge, this is the first study aimed to pre-select serum biomarkers to identify the use of tDCS, and represents the first step toward the development of an indirect strategy, preferably based on the longitudinal monitoring of individual data, for the future detection of “brain doping” in sports.

## Introduction

Transcranial Direct Current Stimulation (tDCS) is a non-invasive and painless brain stimulation technique that involves neuronal excitability and neuronal plasticity through the application of direct current in a specific area of the brain (Truong and Bikson, 2018). This technique was originally developed as a diagnostic tool in neuroscience research, to induce transient and controlled changes in the activity of a specific brain region or network: its application was initially limited to the study of the role of cerebral/sub-cerebral structures in a specific motor, cognitive or perceptual processes. Later on, tDCS has been studied also to explore its utility in the treatment of various neurological disorders, pathologies or syndromes, and it was approved or is presently under approval by the FDA for the treatment of various neurological and psychiatric disorders, including major depression (Berlim et al., 2013), various eating disorders, including bulimia nervosa and anorexia nervosa (Lee et al., 2018), Parkinson disease (Benninger et al., 2010), pain anxiety during burn wound care (Hosseini et al., 2016), chronic pain in multiple sclerosis (Mori et al., 2010) and in spinal injury (Fregni et al., 2006), and also for the improvement of the overall mental state and concentration (Kadosh, 2014).

Apart from its diagnostic and therapeutic utility, tDCS can also represent a potential form of “electromagnetic doping” (or “brain doping”), an innovative concept in the field of performance-enhancing methods in sport (Davis, 2013). However, the list of substances and methods banned by the World Anti-Doping Agency (WADA) (The World Anti-Doping Agency, 2020) does not include this kind of practice, as well as other putative forms of “technological doping,” whose regulation is presently still evolving (for instance, technologically advanced running shoes are allowed, but swimsuits are not). In this context, tDCS sets at the interface between “classic,” pharmacological doping, and “technological” forms of doping.

More in details, “brain doping” consists in the attempt of boosting the sports performance using technologies that may induce a changeset of brain activities through the application of weak direct current that flows between two electrodes on the scalp (Truong and Bikson, 2018). These stimuli can induce a series of effects that can positively impact on sport performance: they include enhancement in muscle strength, attenuation of the sense of fatigue (Okano et al., 2015), reduction of recovery time, and positive changes on mental state and concentration. In particular, anodal stimulation, in which the anode is over the motor cortex, seems to increase sports performance (Banissy and Muggleton, 2013) compared to both the cathodal stimulation and no stimulation condition (Nitsche et al., 2003a), because the excitability of the motor cortex decreases fatigue-related muscle pain (Cogiamanian et al., 2007) and increases motor and perceptual learning (Antal et al., 2004), motivation, and power. Another study determined the effects of anodal tDCS in improving cycling performance, monitoring the increase in time to exhaustion (Vitor-Costa et al., 2015).

Theoretically, the effects of tDCS may resemble those of several different classes of the WADA Prohibited list (see Table 1). Nietsche et al. first showed that weak direct currents are capable of improving implicit motor learning in the human (Nitsche et al., 2003b). In addition, Frazer et al. have shown that four consecutive sessions of anodal tDCS increased cortical voluntary activation, with an improvement in strength, and that the effect appears to be influenced by the brain derived neurotrophic factor (BDNF) polymorphism (Frazer et al., 2016).

**Table 1:** Summary of the reported effects of anodal tDCS and their similarities with those of the classes of prohibited substances presently included in the “Prohibited List” of the World Anti-Doping Agency.

**Effects produced by anodal tDCS**	**Classes of substances of the WADA List showing similar effects**
Increase of attention span	S6
Enhancement of memory and improvement of cognitive ability	S6
Improvement of sport performance through the “motor learning”	S6
Enhancement of muscular strength	S1, S2, S3
Attenuation of fatigue	S1, S4, S7, S9
Increase of endurance time and reduction of recovery time	S1, S2, S4, S6
Induction of changes on mental state and concentration	S1, S6 (S8?)

More recently, the effects of the Halo Sport device, a commercial tDCS system consisting of a headset manufactured by Halo Neuroscience (San Francisco, CA, USA), were tested. Its use was reported to improve repeated sprint cycling power output: 20 min of treatment at 2 mA enhanced the mean power output in cycling sprints, and to have also a positive effect on various aspects of cognitive function, evaluated by the Stroop test (Huang et al., 2019). Positive effect of the Halo Sport device were also reported on the athletic performance of ski jumpers: the skiers' jumping force by 70% and their coordination by 80%, compared with the sham group (Reardon, 2016).

Reported adverse effects are generally mild and reversible, and most of them are mild and disappear soon after stimulation. Recently, it has been shown that tDCS did not induce any major effect on peripheral metabolites (Kortteenniemi et al., 2020). The most common persistent events consist of skin lesions similar to burns, which can arise even in healthy subjects; and mania or hypomania, mostly in patients with depression. The combination of positive effects on the sport performance and the virtual absence of severe side effects makes this technique particularly appealing to attract athletes, especially since the application of tDCS is presently not prohibited. However, to prove the recourse to tDCS is extremely problematic, given the fact that the exact mechanism underlying the stimulation has not been clarified in detail yet. Previous studies have shown that tDCS can promote the inhibition of gamma-aminobutyric acid (GABA), enhance dopamine serum levels (Hadoush et al., 2018) and increase BDNF (Frazer et al., 2016; Hadoush et al., 2018; Cocco et al., 2020), this last effect appearing to be the most significant.

BDNF belongs to the neurotrophins family, which also includes Nerve Growth Factor (NGF), Neurotrophin-3 (NT-3), and Neurotrophin-4/5 (NT-4/5) (Lessmann et al., 2003). These are mature proteins synthesized endogenously from a pro-peptide form (Al-Qudah and Al-Dwairi, 2016). NTs are not only involved in the processes that regulate neurobiology (Pezet and McMahon, 2006), but also in central and peripheral energy metabolism (Pedersen et al., 2009). Particularly, BDNF plays an essential role in the regulation of metabolism in the skeletal muscle, such as fat oxidation and glucose metabolism (Matthews et al., 2012).

A common Single Nucleotide Polymorphism (SNP) rs6265 (G>A, Val66Met), occurs in about 20% of the total world population, with sharp differences among different ethnicities (e.g., 22% in Caucasians, 2% in African Americans) (IGSR: The International Genome Sample Resource, 2021). This polymorphism seems to modulate BDNF secretion and its intracellular distribution, synaptic plasticity, and the negative effect on memory and cognitive function (Chen et al., 2008; Anastasia et al., 2013). Two other SNPs (rs11030099 G>T and rs11030100 C>A), whose occurrence is similar to that of rs6265 (i.e., about 23% of the total world population, 22% in Caucasians, and 5% in African Americans) have been identified in the 3′-UTR BDNF gene to modulate the translation suppression by microRNAs (miRs)-26a and−26b (Caputo et al., 2011).

Based on the above, BDNF seems to be the main neurotrophin implicated in sports performance. Results confirming this idea have already been reported by several research groups (Ferris et al., 2007; Zoladz et al., 2008; Pedersen et al., 2009; Knaepen et al., 2010; Yarrow et al., 2010; Walsh et al., 2016) [reviewed in Walsh and Tschakovsky (2018)].

In the view of potential abuse of tDCS, it would be necessary to identify diagnostic biomarkers representative of the mechanism underlying the stimulation. The most promising biomarker candidates are BDNF, along with its precursor proBDNF, as well as glutamate and dopamine, whose serum levels increase following tDCS. The present study aimed to measure the levels of BDNF, proBDNF and other neurotrophins in serum samples from a cohort of elite athletes, to preliminarily estimate a range of basal concentrations that, due to physical exercise, were expected to significantly differ from those of a population of non-athlete, healthy individuals. Data were also processed to assess any differences based on gender, age, type of sport (power vs. endurance), and type of collection (in-/out-of-competition), to analyze their variability, in view of their selection as putative biomarkers to detect the recourse/abuse of tDCS in sport. Furthermore, the serum levels of BDNF, proBDNF and betaNGF were compared with the levels of some inflammatory markers and growth factors of anti-doping interest in order to verify the extent of a possible correlation aimed to evaluate possible influences on the basal levels of these neurotrophins. Finally, the possible relation between three SNP polymorphisms (rs6265, rs11030099, rs11030100) and BDNF-proBDNF serum values, were also investigated.

## Materials and Methods

### Samples From Elite Athletes

Samples from 92 healthy elite athletes (74 males and 18 females, aged 17–63), of various sports disciplines, tested negative to doping control analysis according to the WADA procedures, were included in the dataset. Samples were selected among those belonging to athletes who had given consent for their samples to be used for research purposes at the moment of sample collection for the doping control test, in agreement with the WADA international standards for laboratories (rev. 2019, appendix A paragraph 2.0) (The World Anti-Doping Agency, 2021). The information reported on the laboratory copy of the doping control form, which is associated to each sample, include sex, type of test (in competition/out of competition), sport discipline, and, in the case the test for human growth hormone is requested, also the age of the athlete. Samples were therefore classified in the following groups: sex (M = 74; F = 18); age (range 17–25 *n* = 27, 26–35 *n* = 36 and over 35 *n* = 11; age unknown *n* = 18); type of sports discipline (endurance = 74; power = 18); and type of test (in competition = 24; out of competition = 68, collected in the following time intervals: 00–08 *n* = 14; 08–12 *n* = 13; 12–18 *n* = 24; 18–24 *n* = 17).

### Serum Samples Collection and Analysis

Blood samples were collected from elite athletes in a BD SST-II gel separator vacutainer after arm venipuncture. Serum samples were obtained after centrifugation at 2,500 rpm for 15 min and stored at −80°C until analysis for NTs concentration using immunoenzymatic, radioimmunological, or flow cytofluorimetric assays. Specifically: Pro-BDNF and BDNF by ELISA Kit Aviscera Bioscience (sensitivity 0.1 ng/mL and 5 pg/mL, respectively, intra-assay precision 4–6% both of them). NT-3 and NT-4 by ELISA kit Sigma-Aldrich (sensitivity 2 and 4 pg/mL, respectively, intra-assay precision < 10%). Mature and biologically active Beta NGF form was assayed by flow cytofluorimetry bead assay R&D systems (sensitivity 0.3 pg/mL, intra-assay precision 6%). Recombinant Human Growth Hormone (recGH, 22 kDa) and Pituitary Growth Hormone (pitGH, 20+22 kDa) by CMZ Assay GmbH (sensitivity 0.03 ng/mL, intra-assay precision 10%). Interleukins IL-8, IL-3, IL-33, Platelet Derived Growth Factor-AA (PDGF-AA), Transforming Growth Factor alfa (TGFalfa), Granulocyte-Colony-Stimulating Factor (G-CSF), Growth regulated protein beta (GROBeta), Human Macrophage Inflammatory Protein-3-beta (MIP3Beta), Epidermal Growth Factor (EGF), and Vascular Endothelial Growth Factor (VEGF) by flow cytofluorimetry beads array (Merck Millipore for Luminex 200, range of sensitivity 0.7–8 pg/mL and intra-assay precision < 5%). Insulin-like Growth Factor 1 (IGF1) and Procollagen III Peptide by radio immunoassays (Immunotech, Beckman Coulter and Orion Diagnostics, sensitivity 40 and 0.7 ng/mL, respectively, intra-assay precision 10%). The optical density and fluorescence readings were measured using an automated microplate reader (Victor3, PerkinElmer, Perkin Elmer Italia Spa, Milano, Italy) and Luminex 200 XMAP (Millipore, Merck Life Science, Milano, Italy); RIA assays were performed on a Wizard 2 (Perkin Elmer Italia, Milano, Italy).

### Genotyping

Genomic DNA was extracted from serum samples using the PrepFiler™ Forensic DNA Extraction Kit (Applied Biosystems, Thermo Fisher) following manufacturer protocols. All subjects were genotyped for rs6265 rs11030099 and rs11030100 BDNF-related polymorphisms. SNP were detected using commercial Taqman Genotyping SNP assays rs6265 C__11592758_10, rs11030099 C__31701071_10 and rs11030100 C__31701070_10) then genotyped on a 7500 Fast Real-Time PCR (Thermo Fisher).

### Data Analysis

Basic statistics was performed by using Statistica 12.0 (Statsoft) and PASW Statistics 17.0 (SPSS-IBM) software packages. Distribution of normality of data was verified with Kolmogorov-Smirnov test. Correlations among analytes was determined by using Pearson and Spearman indexes of correlation (for normally and not normally distributed data, respectively). Mann-Whitney *U*-test was used to confront means among selected group. Statistical significance was set at *p* < 0.05.

## Results and Discussion

### BDNF and proBDNF Serum Concentrations

**Figure 1:**
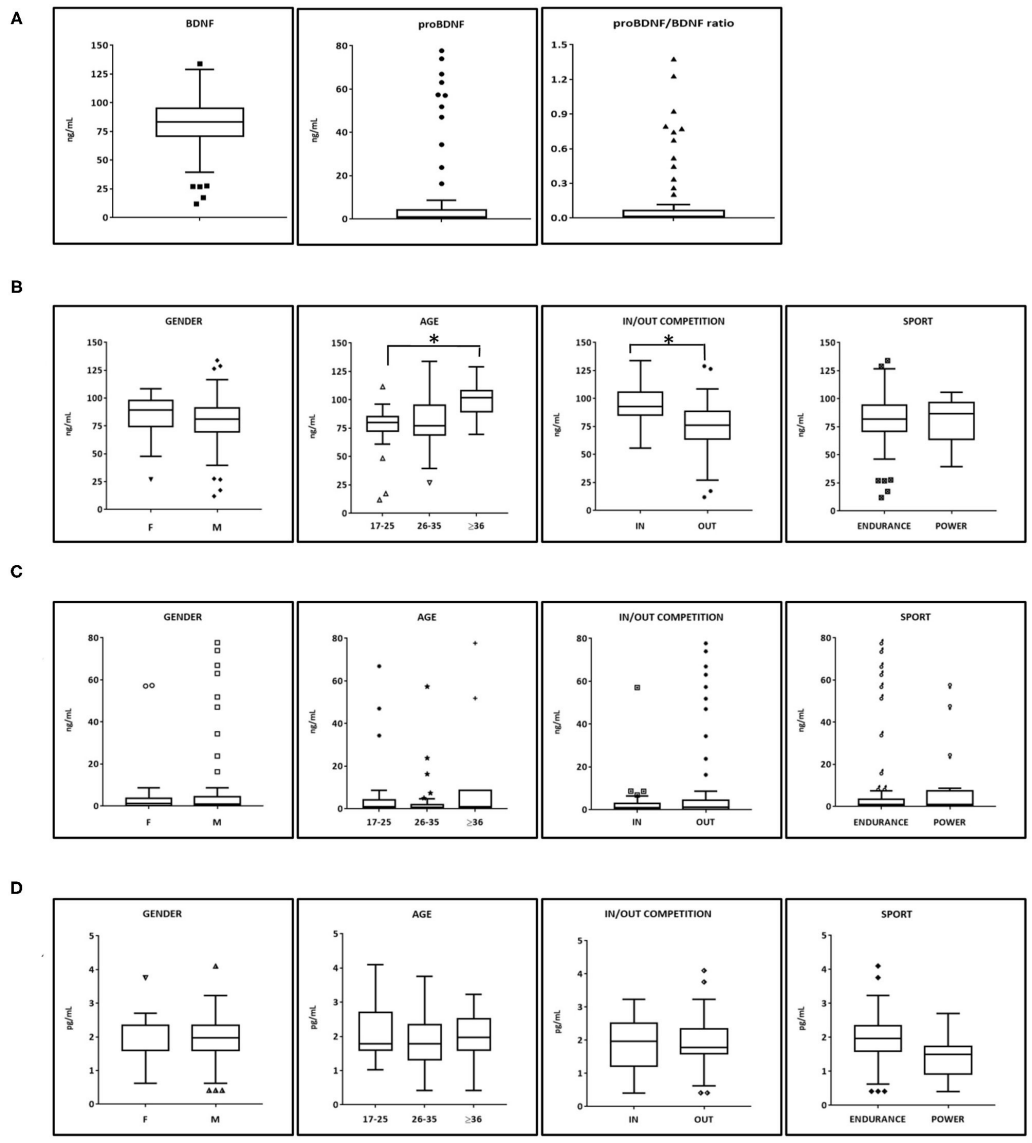
BDNF, proBDNF, and beta-NGF serum levels in athletes. **(A)** BDNF, proBDNF, and their ratio (*N* = 88). **(B)** BDNF serum levels among categories (male *N* = 70, females *N* = 18, Endurance *N* = 70 Power *N* = 18). Age and time of collection show statistical significance as resulted from Mann-Whitney test. **(C)** proBDNF serum levels among categories. **(D)** beta-NGF serum levels among categories. BDNF, brain derived neurotrophic factor; proBDNF, pro-brain derived neurotrophic factor; beta-NGF, beta-nerve growth factor. Symbols outside the box plots indicate the outliers.

Aviscera-Bioscience ELISA kit was chosen for the determination of BDNF and proBDNF assay because of the good performance and the minimal cross-reactivity demonstrated against other NTs or their precursors (Polacchini et al., 2015). Figure 1 shows the serum concentrations of BDNF, proBDNF, and beta-NGF in the whole group of athlete participants (*N* = 92). The BDNF serum concentration was normally distributed with a mean concentration of 80.5 ng/mL (interquartile range 70–100 ng/mL, min 11.9, max 133.9 ng/mL). The distribution of proBDNF (mean 7.7 ng/mL) values was not normally distributed, showing two distinct “subpopulations” of values: one containing subjects with proBDNF concentrations in the range s 0.1–8.0 ng/mL (*n* = 76), and 16–80 ng/mL (*n* = 12), respectively. 11 out of 12 samples with higher proBDNF level were collected out of competition, equally distributed for each 24 h time interval (00.00–08.00 h: *n* = 2; 08–12.00: *n* = 2; 12.00–18.00: *n* = 3; 18.00–24.00: *n* = 4). Consequently, high proBDNF values cannot be associated to the state of rest (samples collected in the time interval 00.00–08.00).

A low negative linear correlation was found between BDNF and proBDNF (Pearson *r* = −0.24 *p* < 0.05, Spearman ρ = −0.13 but *p* > 0.05). Higher correlation was found between BDNF and proBDNF/BDNF (*r* = −0.40, *p* < 0.01 and ρ = −0.31, *p* < 0.01). beta NGF does not significantly correlate to BDNF nor to proBDNF.

Subsequently, the database was analyzed according to the gender of athletes, their age, the time of blood withdrawal (IN competition at the end of a sports event or OUT competition during rest or training), and the type of sport practiced (power vs. endurance). A statistical significance emerged in athletes older than 36 years with respect to the group aged 17–25 years old but not compared to the athletes aged 26–35 years old. Another statistical significance emerged in BDNF values from samples collected IN competition (*N* = 26), and BDNF values obtained OUT of competition (*N* = 66).

The distribution of proBDNF values according to the four subcategories analyzed shows no statistically significant differences.

Beta-NGF resulted in being detectable in all serum samples (range of values 0.40–4.10 pg/ml, mean = 1.94; median = 1.97 pg/ml). No statistically significant differences emerged in the subdivisions among categories. As regards the other Neurotrophins, NT-3 and NT-4 were only detectable in a small number of samples (10%) most likely because they are present at a level below the sensitivity of the immunological method (2 and 4 pg/mL, respectively).

BDNF serum levels of elite athletes obtained in this study have been then compared to those from healthy non-athletes (sedentary or non-trained individuals) obtained from literature and assayed by immunological methods similar to those used in this study. The mean serum BDNF for non-athletes aged in the range <20, 18–29, and 31–55 resulted in 20.9, 24.4, and 29.3 ng/mL, respectively (Elfving et al., 2012; Hötting et al., 2016; Kim, 2016). All these values are well lower than those we found among elite athletes (Figure 1B) for any age category. We may conclude that competitive sport activity results in an increase of BDNF basal serum levels, and this is especially true at the end of a competition. Traumatic factors at both muscular and psychological levels (such as stress or anxiety) may be the cause of such an increase.

### SNP Genotyping and Haplotype Analysis

**Figure 2:**
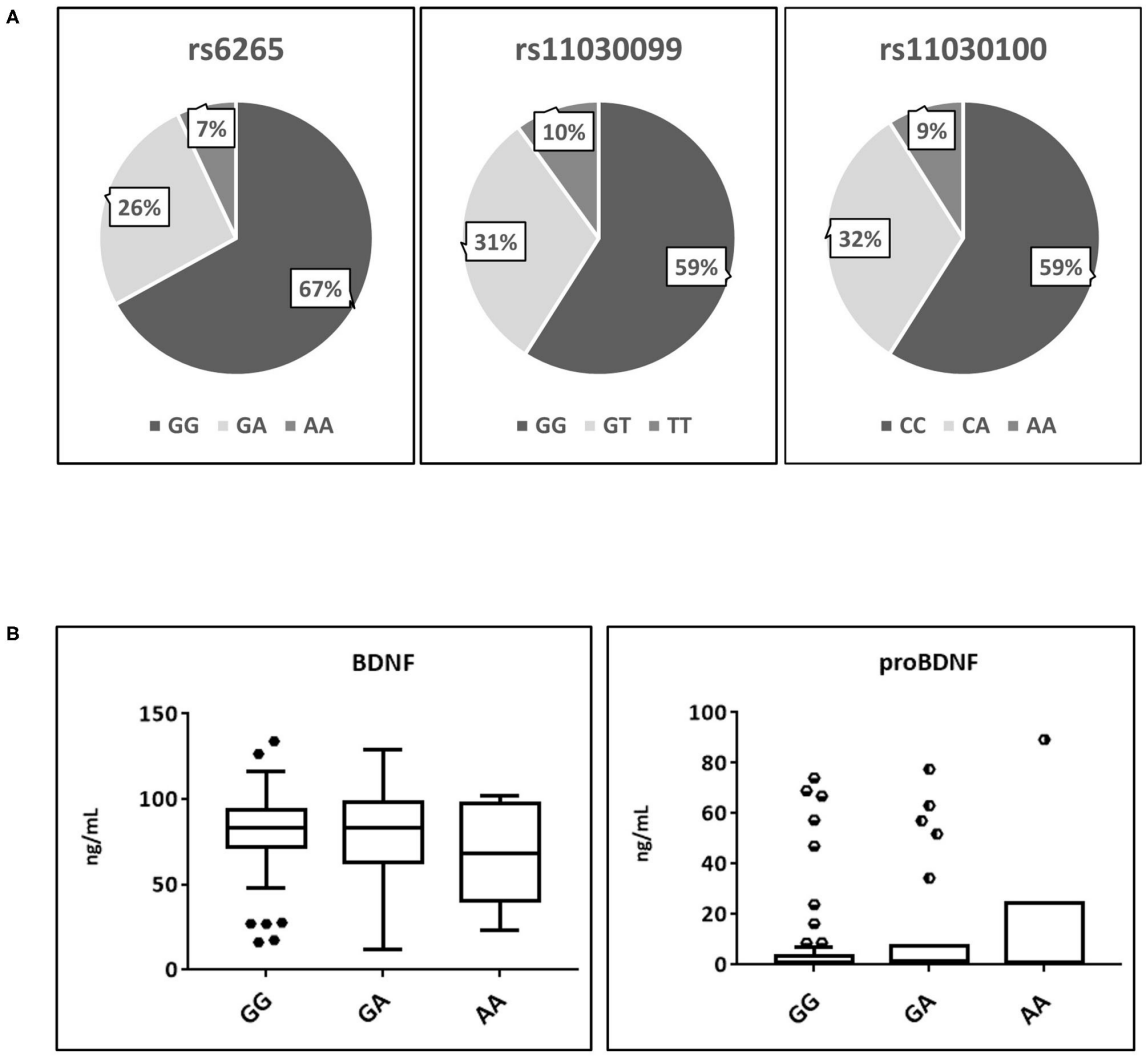
Analysis of the main polymorphisms affecting proBDNF and BDNF secretion. **(A)** Frequency percentages of the three polymorphisms. **(B)** BDNF and proBDNF serum distribution affected by rs6265 polymorphism. BDNF, brain derived neurotrophic factor; proBDNF, pro-brain derived neurotrophic factor. Symbols outside the box plots indicate the outliers.

A total of 88 athletes of the database have been genotyped for SNP rs6265, rs11030099, and 11030100. Figure 2A shows a very similar situation in the frequency percentages of the three genotypes, confirming the strong linkage between these genetic systems, especially for rs11030099 and rs11030100. All these genetic systems respect the Hardy-Weinberg equilibrium as verified by the Chi-Squared test (data not shown).

As shown by Figure 2B, no significant differences were found in the serum levels of both BDNF and proBDNF regards the three possible genotypes of rs626, where the allele frequencies (*G* = 77.8% *A* = 17.6%) nearly overlapped those reported for the global population (Matthews et al., 2012).

**Figure 3:**
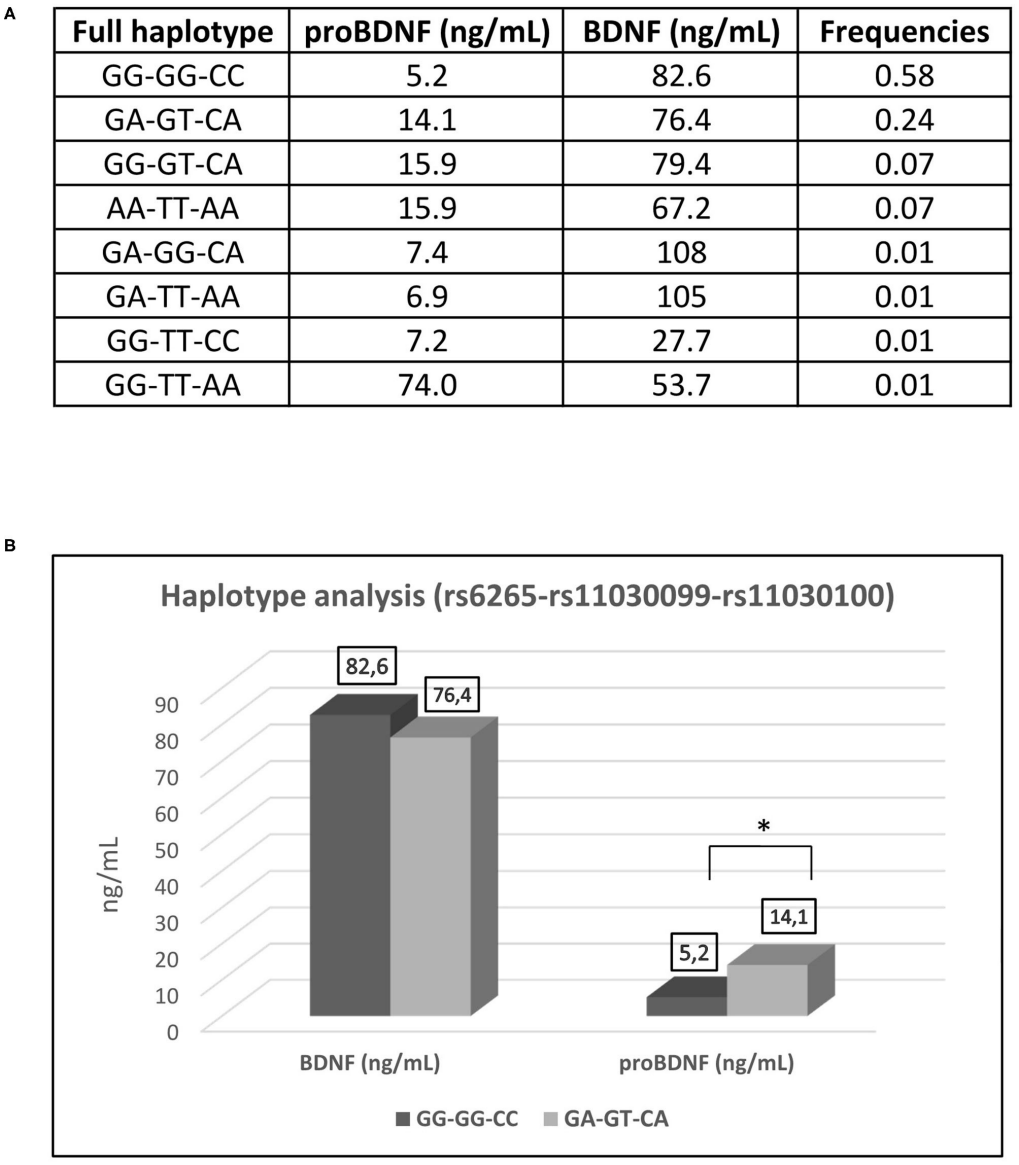
Haplotype analysis of the SNP (rs6265-rs11030099-rs11030100). **(A)** Frequencies of haplotypes. **(B)** Haplotype analysis of GG-GG-CC (full wildtype haplotype) vs. GA-GT-CA (full heterozygous haplotype) in regard of serum concentration of BDNF and proBDNF. BDNF, brain derived neurotrophic factor; proBDNF, pro-brain derived neurotrophic factor. *p < 0.05.

A wider haplotype analysis (Figure 3A) showed GG-GG-CC as the most common haplotype (58%). This refers to individuals who are homozygotes for the ancestral alleles of all the three SNP (rs6265-rs11030099-rs11030100, respectively). Interestingly, the second most common haplotype (24%) refers to individuals that are heterozygous for the three SNP (GA-GT-CA). These individuals share one ancestral allele and one polymorphic allele. A full homozygous polymorphic haplotype (AA-TT-AA) was found in 7% of the individuals. Other haplotypes were found to be present at 7% or less.

The three SNPs are linked together as showed by their relative frequencies and despite the fact that they exert effects on different sites of the BDNF precursor. Athletes from both “in competition” and “out of competition” groups, who are full heterozygous for the haplotype GA-GT-CA present higher and statistically significant proBDNF values than athletes carrying the full ancestral genotype GG-GG-CC (Figure 3B). This can be coherently explained by the fact that the presence of the polymorphism determines the suppression of the recognition site for the miRNAs acting on the 3′-UTR of the BDNF precursor mRNA. Very high proBDNF values are attributable to the presence of the polymorphisms rs11030099 and rs11030100 (indifferently if they are present at the homozygous or heterozygous state) rather than to the presence of the polymorphism rs6265. Curiously and interestingly, the same pattern is not observed for the serum values of the BDNF, probably because BDNF may follow a different dynamic from proBDNF once it is released into the systemic circulation. The difference recorded between the values of proBDNF and BDNF could also be due to a BDNF reservoir contained within the circulating platelets (Walsh and Tschakovsky, 2018).

**Figure 4:**
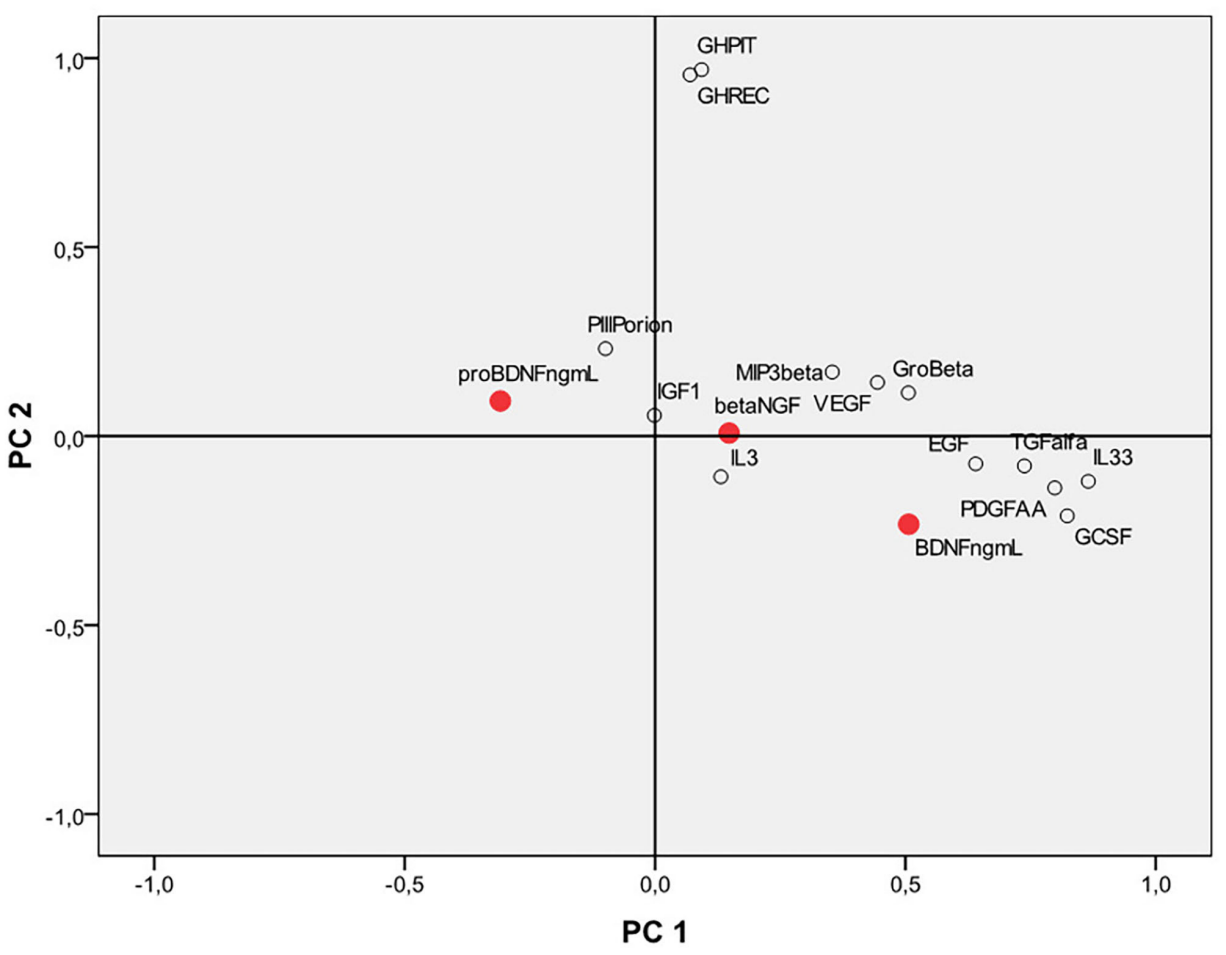
Principal component analysis (PCA) considering the neurotrophins BDNF, proBDNF, beta NGF and other growth factors, and human inflammation markers. BDNF, brain derived neurotrophic factor; proBDNF, pro-brain derived neurotrophic factor; beta-NGF, beta-nerve growth factor.

Principal Component Analysis (PCA) was performed to evaluate possible correlations between the neurotrophins and some other markers (Figure 4). In particular, we focused both on markers related to the state of inflammation and stress (eg PDGF-AA, IL8, IL3, IL33, TGFalfa) and on other markers already routinely monitored in anti-doping analysis such as IGF-1, P-III-P, human growth hormone in its two major isoforms, namely recombinant GH (recGH, 22 kDa) and pituitary GH (pitGH, 20+22 kDa).

Interestingly, BDNF correlates and clusters together with IL-33, PDGF-AA, TGF alfa, and G-CSF in an area of the first main component that seems to be mostly related to the family of inflammation markers rather than on the second component, which seems to be mostly related to the family of skeletal growth factor markers (mainly represented by GH).

ProBDNF is located in an opposite position on the plot and does not cluster with inflammation markers nor with the skeletal growth factors. Beta-NGF is in an intermediate position between the proBDNF and the BDNF. No high values of linear correlation were found for BDNF, proBDNF, and beta-NGF compared to the other analytes. Apart from the modest negative correlation found with proBDNF and BDNF, other statistically significant correlations have been found only between BDNF vs. IL-33, PDGF-AA, and TGF alfa (*r* = 0.33, 0.40, and 0.36, respectively). proBDNF also significantly correlates (negatively) with PDGF-AA (*r* = −0.32). beta-NGF does not significantly correlate (all *r*-values smaller than ±0.2) with any of the markers confronted. Further studies on a larger population of subjects are however necessary to draw more solid conclusions.

## Conclusions and Future Perspectives

An exploratory analysis was conducted here in order to study the baseline variability of neurotrophins in a group of elite athletes. Data collected in this study suggest the possible selection of BDNF as the principal putative biomarker to detect the recourse to tDCS in sport; while the diagnostic utility of proBDNF, and beta-NGF need to be further assessed. BDNF, proBDNF, and their ratio are relatively stable in time, although variability between subjects has been ascertained, especially for proBDNF. Statistically significant differences were found in the following cases: (1) elite athletes older than 35 years old showed higher BDNF values than younger subjects, (2) samples collected IN competition presented higher BDNF concentrations than those collected OUT of competition. The three polymorphisms analyzed seem to have an effect only on proBDNF and do not influence BDNF serum concentrations. The adoption of population-based thresholds does not seem of utility to detect the application of tDCS. Due to their high inter-individual variability, BDNF and proBDNF can be selected as putative biomarkers to detect the recourse to tDCS in sport, by monitoring their levels longitudinally for each subject: this would allow to obtain individual normality ranges, as in the Athlete's Biological Passport (ABP). Future studies are being designed to assess the intra-individual variability of the putative markers considered in this study, as well as to identify additional potential biomarkers of brain doping, starting from cellular models and ending to human subjects, also by proteomics approaches. PCA shows that markers of inflammation (such as pro-inflammatory cytokines) and growth factors (such as PDGF) seem to be the most promising candidates to be included in future studies.

Limitations of this study are the relative low number of elite athletes (limited to those who had expressed their consent to the use of their sample for research study on the doping control form) and the lack of detailed information for each subject (doping control samples are rigorously anonymous, and the age of the subjects is not reported in all cases).

Ultimately, the relevance of BDNF and proBDNF as biomarkers of tDCS could be finally confirmed by measuring their levels in volunteers before and after treatment by tDCS itself. Nonetheless, to the best of our knowledge, this is the first study aimed to analyze the possibility of using serum biomarkers to identify the potential abuse of tDCS among athletes. It also represents the first essential step toward the development of an indirect analytical strategy for the future detection of “brain doping” in sports.

## Data Availability Statement

The original contributions presented in the study are included in the article/supplementary material, further inquiries can be directed to the corresponding authors.

## Ethics Statement

The studies involving human participants were reviewed and approved by Compliant with the International Standard for Laboratories of the World Anti-Doping Agency (WADA-ISL), allowing the use of biological samples from official doping control test, provided the athlete has authorized the use of his/her sample for research purposes by ticking the relevant field on the doping control form and the samples - already anonymous - are also re-coded. The patients/participants provided their written informed consent to participate in this study.

## Author Contributions

FD: conceptualization, project administration, method development, writing – review and editing, data analysis, and supervision. VS: methodology, validation, original draft preparation, and data curation. GB: original draft preparation, data curation, and reviewing and editing. XT: conceptualization, reviewing and editing, supervision, and data analysis. FF: conceptualization, project administration, reviewing and editing, and supervision. FB: conceptualization, funding acquisition, project administration, reviewing and editing, supervision, and submission. All authors contributed to the article and approved the submitted version.

## Conflict of Interest

The authors declare that the research was conducted in the absence of any commercial or financial relationships that could be construed as a potential conflict of interest.
